# A Potential Pitfall in the Interpretation of Microscope-Integrated Fluorescence Angiography: The Center–Periphery Effect

**DOI:** 10.3390/medsci13020054

**Published:** 2025-05-03

**Authors:** Dieder Stolk, Paul Bloemen, Richard Martin van den Elzen, Martijn de Bruin, Caroline Driessen

**Affiliations:** 1 Department of Vascular Surgery, Noordwest Ziekenhuisgroep Alkmaar, 1815 JD Alkmaar, The Netherlands; 2 Department of Biomedical Engineering and Physics, Amsterdam Universitair Medische Centra, Meibergdreef 9, 1105 AZ Amsterdam, The Netherlands; p.r.bloemen@amsterdamumc.nl (P.B.); d.m.debruin@amsterdamumc.nl (M.d.B.); 3 Department of Plastic, Reconstructive and Hand Surgery, Amsterdam Universitair Medische Centra, Meibergdreef 9, 1105 AZ Amsterdam, The Netherlands; c.driessen@amsterdamumc.nl

**Keywords:** near-infrared imaging, fluorescence angiography, indocyanine green (ICG), microscope

## Abstract

Background/Objectives: Indocyanine green fluorescence angiography (ICG-FA) enables the real-time visualization of tissue perfusion. However, objective research on microscope-integrated fluorescence angiography (FA) has not been conducted before. This study aims to evaluate the fluorescence light distribution in images formed by ICG-FA in two surgical microscopes using a phantom, and to provide recommendations for their application. Methods: An 11.8 by 6.8 cm ICG and Intralipid phantom was made to evaluate overall spatial fluorescence sensitivity in two surgical microscopes in multiple working distances (WDs) and magnification factors (MFs). The signal was quantified using a tailor-made software in Python 3.8.10. Results: A clear center–periphery effect was present in most settings in both microscopes, with the highest peripheral fluorescence signal loss in the lowest MF: 100% in the Tivato and 83% in the Pentero. Increasing the MF improved homogeneity, where the biggest difference was seen between the first and second MF. A 30 cm WD and 3.5× MF produced the most homogeneous images suitable for free-flap surgery. Manually opening the light beam diameter also reduced the center–periphery effect. Conclusions: Peripheral signal loss in microscope-integrated ICG-FA must be considered during clinical interpretation and for the quantification of tissue perfusion. In clinical practice during reconstructive free-flap surgery, a 30 cm WD, 3.5 MF, and manually opened light beam diameter should be applied to achieve the most homogeneous image.

## 1. Introduction

Near-infrared fluorescence (NIRF) imaging using Indocyanine green (ICG) is an advanced medical imaging technique widely implemented in surgery for its ability to facilitate real-time information about tissue perfusion [1,2,3,4,5]. In reconstructive surgery, ICG fluorescence angiography (ICG-FA) is mainly used to evaluate the viability of skin flaps intraoperatively [5,6,7,8]. Moreover, it is sometimes used to verify the patency of the anastomoses, and it could possibly also be used to facilitate the decision-making of excising the less viable zones of a flap [9,10]. One study showed that debridement after ICG-FA allowed a tenfold reduction in the occurrence of fat necrosis compared to a standard procedure [5].

Fluorescence is defined as the emission of radiation or light after having absorbed radiation from another source [11]. ICG is a commonly used water-soluble fluorescent dye that is only visible in near-infrared light and has a peak absorption of 805–810 nm and peak emission of 835 nm [10]. After intravenous administration, ICG binds efficiently to plasma proteins, making it a reliable indicator of vessel perfusion [12]. Its high sensitivity and low toxicity aids in the widespread use of ICG [13,14].

In addition to providing visual assistance, ICG fluorescence angiography (ICG-FA) has great potential to quantify tissue perfusion [15,16,17]. However, the interpretation of the fluorescence signal remains subjective, as images of the same target may vary considerably when acquired from different camera systems. This complicates standardization and accurate interpretation for clinicians.

**Figure 1:**
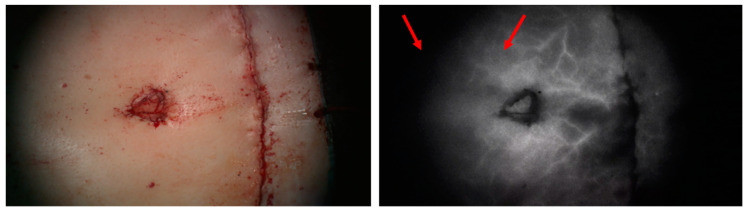
Image of the umbilicus and closed abdominal donor site during a DIEP procedure, using the Zeiss Tivato 700, Meditec AG, Jena Germany, in white light (**left**) and during ICG-FA (**Right**). A clear difference in fluorescence signal intensity can be observed between the two areas highlighted by the red arrows.

The type of imaging system used is dependent on availability, hospital contracts with businesses, and personal preference. Researchers have tried to compare systems by assessing overall fluorescence sensitivity, resolution performance, ideal distance from the object, and other factors known for influencing ICG-FA output [18,19,20]. A recent study provided new insights on the concept of the heterogeneity of signal sensitivity within NIRF systems. Joosten et al. showed variability in light distribution in different laparoscopic and hand-held ICG-FA systems, with a strongly diminished fluorescence sensitivity in the periphery of the field of view (FoV) [21]. This has never been researched for surgical microscopes but could explain why a similar image is seen in clinical practice when using a microscope for ICG-FA, as shown in Figure 1. Although the two areas highlighted by the red arrows are expected to be equally perfused, the fluorescence signal in the periphery is noticeably reduced. However, this would be corrected after relocating the same area to the center of the FoV.

Whereas certain NIRF camera systems are developed specifically for ICG-FA, the primary function of microscopes in the operating room is magnifying an area of interest to enable precision in microsurgery. It would be highly advantageous to be able to utilize additional functions such as fluorescence, given that it would not require any supplementary equipment. This would improve the user-friendliness for clinicians and yield benefits in terms of cost and time efficiency. Research has shown ICG-FA by microscopes to be valuable intraoperatively, but an objective quality assessment has not been executed yet [22,23].

We hypothesize the center–periphery effect that Joosten et al. describes to also be present in microscopes. Furthermore, magnification might influence this considerably. In a surgical microscope, different consecutively placed lenses coact to achieve magnifications [24]. Due to high angles in light waves when using a low magnification factor (MF), there is a lot of loss of light in the periphery of the view, also called vignetting. When increasing magnification, the angles of the light waves decrease, thus reducing the center–periphery effect. Since the software in each system can also influence the eventual image shown, research on the output of the microscopes is crucial.

To investigate the center–periphery effect in surgical microscopes, we evaluate the measured light distribution of two microscopes (Zeiss Meditec AG, Jena, Germany) by using a phantom and the impact of focal distance and magnification. Secondly, we intend to use the data to make recommendations on the implementation of the microscopes for reconstructive surgery.

## 2. Materials and Methods

In this study, the microscopes Zeiss Tivato 700, Carl Zeiss Meditec AG, 2019, Jena Germany, and the Zeiss OPMI Pentero 900, Carl Zeiss Meditec AG, 2021, Jena Germany, were evaluated in an experimental setting in the Amsterdam UMC.

### 2.1. Phantom

A homogeneous fluorescent phantom was created by combining Intralipid (IL) 20%, agarose, ICG, and phosphate-buffered saline (PBS) in a polypropylene container. The inner dimensions of the container were 16.8 × 11.8 cm and 6.8 cm in height and contained a total of 0.8 L phantom. First, 400 mL PBS and 16 g agarose were mixed and heated to produce a 2% agarose dilution. Then, 400 mL IL was mixed with 365 microliters ICG. These two liquids were combined to form phantom 1. For the calculation of ICG volume percentages in the phantom, a dosage of 0.1 mg/kg bodyweight was used, following Amsterdam UMC guidelines, with the assumption that a patient of 80 kg contains 7 L of blood [25]. For verification of the data, a second phantom was created with half the dosage, 182 microliters ICG.

### 2.2. Fluorescent Assessment

**Figure 2:**
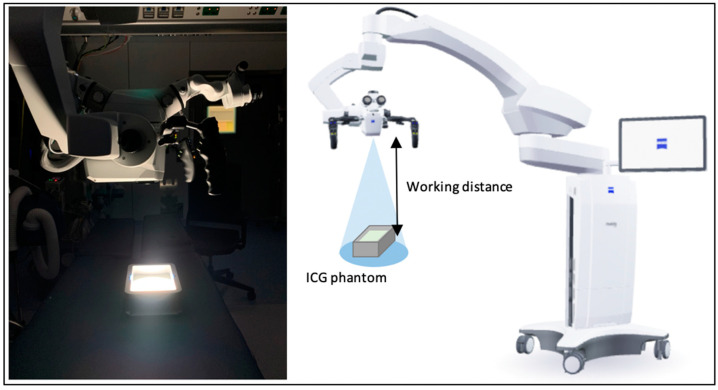
Experimental set up for both microscopes. The surrounding lights were dimmed to mimic fluorescence angiography conditions and constant settings were used: light intensity 50%, gain 0%, excitation spot open.

The two microscopes were tested in an operating room in the Amsterdam UMC, with dimmed lights to simulate fluorescent angiography conditions. The phantom was placed on the operating table with the microscope positioned above it at a 90-degree angle, see Figure 2. Full High Definition, 8-bit video recordings (1920 × 1080 pixels) were made at a working distance (WD) of 20, 30, 40, and 50 cm from the phantom, as indicated by the microscope. For the Tivato 700, recordings were also made at 60 cm due to a greater reach in the camera. For each WD, recordings of 10 s were made at minimal, 3.5×, 5.5×, 7.5×, 10×, and maximum magnification, of which the minimal and maximum magnification factor (MF) varied between microscopes and each of the WDs. Light intensity was set to 50%, gain to 0%, and the illuminated spot was manually set to the largest field of area view. Additionally, some underexposed images using the Tivato were also recorded at a light intensity of 100% and a series of extra recordings were made without manually opening the spot. A gain setting of 0% was applied to minimize the influence of model-specific software and to ensure consistency across all measurements. Dark measurements were taken for both systems. The recordings were made on two consecutive days; the Pentero 900 was tested on day one and the Tivato 700 was tested on day two.

### 2.3. Analysis

Special software written in Amsterdam UMC using Python programming language (Python Software Foundation 3.8.10) was used for the quantitative analysis of the recorded video sequences. The software formed a grid on the recording and then measured the mean fluorescence intensity in each cell as an arbitrary unit from 0 to 255. Grids consisting of 28 × 16 cells and 25 × 20 cells were used for the Tivato and Pentero, respectively. To account for minor disruptions in captured light, an output of 15 consecutive frames per recording was averaged for the final analysis.

To assess the homogeneity of the FoV, the coefficient of variation (CV) was calculated by the following:

$$CV=\frac{\sigma }{\mu },$$

in which σ indicates the standard deviation and μ indicates the mean of the values of the different cells [26]. A decrease in the CV will illustrate a more homogeneous FoV. The percentage of maximum fluorescence signal loss (*FSL*) in the FoV was calculated by the following:

$$FSL=(1-\frac{F_{low}}{F_{high}})\times 100\%$$

in which *F_low_* indicates the lowest fluorescent intensity and *F_high_* indicates the highest fluorescent intensity. An increase in FSL signifies a greater difference in signal intensity between the cell with the highest intensity and the cell with the lowest intensity. The absolute fluorescence intensity in each cell was calibrated to develop a 2D overview of light distribution.

To evaluate the inter-rater reliability of the CV data within the instruments and between the first phantom and second phantom, the intraclass correlation coefficient was calculated using SPSS Statistics 29.0 [27].

### 2.4. Photobleaching

After the experiments with both systems, we performed a photo bleaching test by exposing the phantom during 12 min on 100% light intensity and recording the fluorescent signal every 3 min to analyze the decay in the intensity of the recorded images.

## 3. Results

### 3.1. Light Distribution

**Figure 3:**
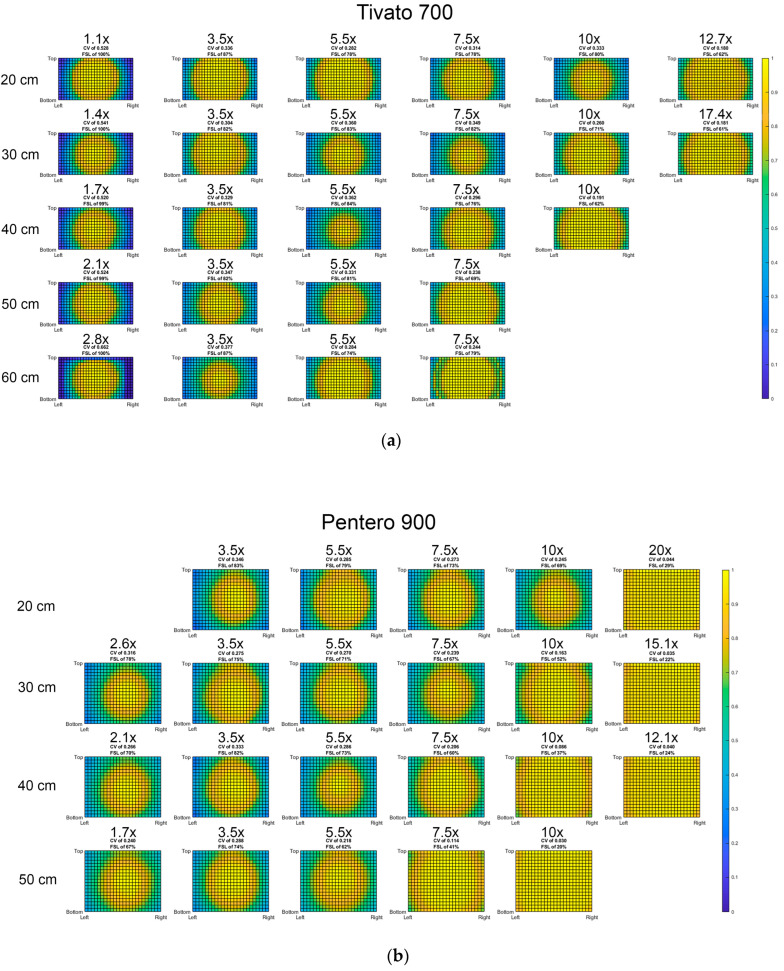
Light distribution per setting in (**a**) the Tivato 700 and (**b**) Pentero 900. Relative fluorescence intensity portrayed in the colors: yellow for maximum (1.0) intensity, until dark blue (0.0) for zero intensity. Each grid represents a different setting: focal distance increases from top to bottom, portrayed in cm. Magnification factor increases from left to right. For each setting, coefficient of variation (CV) and maximum fluorescence signal loss (FSL) are also shown.

The lowest CV scores, indicating the best homogeneity of the FoV, were observed at the highest magnification factors (MFs), irrespective of working distance. The CV, FSL, and an illustration of the normalized light distribution within the field of view (FoV) for each setting is presented in Figure 3, where the color yellow represents the highest intensity, and dark blue represents the lowest intensity. The Pentero microscope exhibited overall lower CV scores than the Tivato, with a range of 0.36 to 0.03 (Pentero) and 0.66 to 0.18 (Tivato). The Tivato also showed higher FSL scores compared to the Pentero, with FSL scores of up to 100% at the lowest MF. In the Pentero, the highest FSL was 83% at the lowest MF at 20 cm WD.

A FoV suitable for free-flap perfusion assessments was obtained with a MF up to 3.5: 75 × 61 cm in the Pentero and 67 × 37 cm in the Tivato (Table A1, Appendix A). All FoVs produced by a higher MF than 3.5 were deemed too small to efficiently assist in the free-flap assessment. Within the range from MF 1.1 to 3.5, the lowest CV and FSL combination was seen at 30 cm WD and 3.5× MF in the Tivato and 50 cm WD and 1.7× MF in the Pentero.

**Figure 4:**
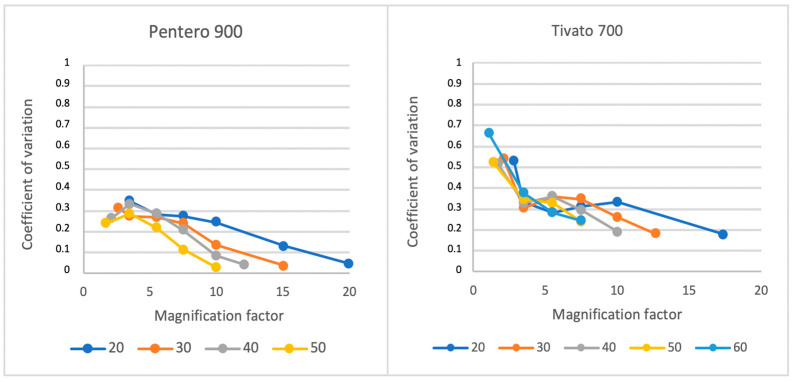
Variability in homogeneity (CV) in the Pentero 900 and Tivato 700 per working distance (cm) from the phantom.

A graphical representation of the CV trend with respect to magnification for each working distance is presented in Figure 4. For the Tivato, the largest drop in CV can be seen from a minimal magnification until 3.5× magnification in each WD. This phenomenon could not be seen for the Pentero. The Pentero, however, did display a decrease in CV when increasing the WD, but only for an MF of 5.5× and higher.

### 3.2. Influence of Light Beam Diameter

**Figure 5:**
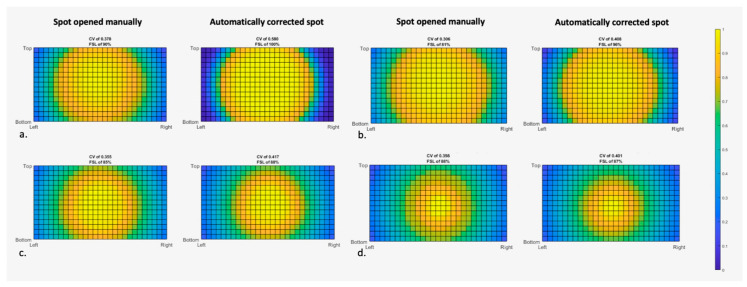
Influence of light beam diameter on light distribution in the Tivato 700 using phantom 2. The recordings were made with a magnification factor of 3.5× and working distance of (**a**) 20 cm, (**b**) 30 cm, (**c**) 50 cm, and (**d**) 60 cm. Coefficient of variation (CV) and fluorescence signal loss (FSL) per setting are shown, as is the relative light intensity, portrayed in the colors: yellow for maximum (1.0) intensity, until dark blue (0.0) for zero intensity.

Figure 5 shows the impact of beam size on light distribution in the Tivato. As seen in lower WDs, manually opening the spot resulted in a decrease in CV by at least 0.1. The effect became less pronounced as the WD was increased.

### 3.3. Absolute Maximum Light Intensity

Table 1 and Table 2 demonstrate the absolute maximum light intensity (AMI). The data are adjusted to the baseline output during dark measurements: 0 for the Tivato and 22 for the Pentero. The AMI decreases strongly in the highest MF and WD, especially in the Tivato. The Tivato exhibited a maximum reduction of 97% while increasing WD (214 to 6); the Pentero showed a maximum reduction of 69% (135 to 42).

An especially strong decrease in AMI is seen when using the Tivato at 60 cm, where the AMI is close to 0 in the highest magnification factor. To improve the AMI, the excitation light intensity was manually increased from 50% to 100% in the most underexposed images in the Tivato 700, which caused an increase in AMI of more than 270% in all recordings. The results of this analysis are shown in Table 3. This also resulted in a clear decrease in CV. The extent of this reduction seemed dependent on the starting value at 50% light intensity: the lower the AMI at a 50% light intensity, the bigger the difference in CV compared to the 100% light intensity recording. Starting values under 25 all showed a reduction of more than 10% in CV.

### 3.4. Reliability Analyses

The intraclass coefficient between the two phantoms per microscope was 0.992 (95% CI: 0.979–0.996) for the Tivato 700, and 0.989 (95% CI: 0.974–0.995) for the Pentero 900. The overall intraclass coefficient between the two phantoms was 0.992 (95% CI: 0.986–0.996). During the photobleaching test, a signal intensity loss of 3% was observed.

## 4. Discussion

Investigating image quality in NIRF systems is crucial not only to ensure the accuracy of image interpretation by physicians, but also to enable data quantification and interchangeability between systems. This study is the first to examine the center–periphery effect during fluorescence imaging in surgical microscopes (Tivato 700 and Pentero 900, Zeiss Meditec AG, Jena, Germany) by using a phantom. First of all, the fluorescence function of the microscopes proves valuable in clinical practice. However, clinicians should be aware of the limitations in the FoV, as MFs up to 3.5 were deemed suitable for free-flap assessments; all FoV’s using higher MFs were considered too small. Secondly, the results show great variability in light distribution among different settings, but a clear center–periphery effect, a reduction in fluorescence intensity at the edges of the FoV, was seen in almost all recordings. This negative effect may be explained by (1) light intensity dependence on the angle of the light beam, (2) the imperfect alignment of the optical system (lenses, mirrors etc.), (3) the shape of the light cone, and (4) lens characteristics.

These observations are most likely relevant to near-infrared fluorescence (NIRF) imaging with microscopes in general, given the similarities in set up and technique. The center–periphery effect is likely linked to the interaction between the illuminated field and the FoV projected onto the camera sensor. Specifically, when the illuminated spot is smaller than the field captured by the optics, peripheral regions receive less excitation light, leading to a lower fluorescence signal. This effect is inherent to optical systems that use converging lenses and illumination cones that do not fully cover the entire imaging area uniformly. Such optical limitations are not specific to a particular microscope model or manufacturer but are influenced by general principles of optical design, such as the configuration of the objective lens, magnification optics, working distance, and even the software algorithms used for image processing. Therefore, these findings should be seen as applicable to all microscope-based ICG-FA systems, rather than being limited to the Tivato 700 or Pentero 900. Future research should aim to validate the observations made across a wider range of microscope systems and also exoscopes to better characterize system-agnostic best practices for intraoperative fluorescence imaging.

In both systems, the highest magnification settings produced the most homogeneous images. The Pentero 900 showed an overall better image homogeneity compared to the Tivato 700 and a stronger correlation between magnification and working distance. In MFs bigger than 5.5, increasing the working distance improved the homogeneity, but a bigger MF is clinically less useful due to a limited field of view. For the smaller MFs, the working distance does not improve homogeneity. Depending on the desired FoV, different recommendations can be made on application. Although the most homogeneous image was observed at 50 cm WD and 1.7× MF within the range of FoV suitable for free-flap evaluation, a more useful image could be obtained at 30 cm WD and 3.5× MF due to a much higher absolute intensity. However, it is important to note that the user should still consider a 75% maximum signal loss at the periphery compared to the edges.

The lack of correlation between magnification and working distance implies that the software has a more significant impact on the resulting image in the Tivato 700. Although the Tivato could achieve greater working distances from the object (60 cm versus 50 cm in the Pentero), a drastic reduction in absolute light intensity (AMI) was observed, potentially rendering the resulting image unsuitable for clinical use. Notably, the lowest MF produced a substantially inferior image compared to an MF of 3.5, with a 100% signal loss at the periphery relative to the center. The optimal choice for reconstructive surgery would be 30 cm WD with a 3.5× MF. Whereas software, lens characteristics, and optical alignment cannot be adjusted by the user, an awareness of these limitations is essential to ensure the reliable interpretation of fluorescence images. Further improvement in image homogeneity can be achieved by increasing the diameter of the light beam, improving the FSL from 96% to 81%. This can be explained by the reduction in light angle dependence, as a broader light beam results in a more uniform angle of incidence across the FoV.

Consistency in homogeneity should be maintained regardless of the excitation light intensity. The considerable contrast in the coefficient of variation (CV) between images captured at 50% and 100% excitation levels signifies that the data obtained from underexposed images are less reliable. The bigger the difference in the CV, the lower the reliability of the results. This effect can be seen particularly at intensities below 25. Nevertheless, these findings do not affect our ability to make recommendations for clinical practice, since such low intensities were observed only in the smallest FoVs. We did find a high level of reliability within instruments and across phantoms.

Recent research has focused on the quantification of tissue perfusion, but this is complicated by the significant variability in images among the systems utilized [17]. Various factors are known to impact the image formed during ICG-FA. Research on the ideal working distance, center–periphery effect, and absolute light intensity has been conducted for laparoscopic and hand-held NIRF systems [20,21], but this study is the first to offer objective outcomes regarding the NIRF images produced by surgical microscopes. It is crucial to take the center–periphery effect into consideration, not only in clinical practice but also with the aim of quantification. The data of this study can be used to provide recommendations for microscope usage, but they also have potential for correcting NIRF images to enable reliable quantification, as was concluded for other systems recently by Joosten et al. [21]. Theoretically, even the real-time correction of fluorescence images during surgery could become possible. However, applying such corrections in practice remains a major challenge, as living tissue presents a complex, three-dimensional surface—unlike the flat, uniform geometry of a phantom—making accurate calibration and compensation significantly more difficult.

In this study, surgical microscopes performed worse in terms of FSL when compared to some hand-held and laparoscopic systems in previous research. Our findings suggests that surgical microscopes are more prone to signal intensity loss at the edges of the image compared to hand-held and laparoscopic devices [21]. However, FSL alone does not provide a complete picture and only focuses on one cell in the grid, which makes it difficult to compare image efficiency. This is particularly relevant for free-flap assessments, where the entire screen is used to capture overview images in contrast to laparoscopic surgery, where the focus lies on a specific area of interest in the center.

Being able to use surgical microscopes for both creating anastomoses and fluorescence angiography of the skin flap would be highly advantageous, as it eliminates the need for an additional camera system. It may also be useful in other fields of plastic surgery, such as studying the vascularity of palatal flaps in cleft surgery, which is also more commonly performed with the use of the microscope. However, when an overview of a large flap is required, the position of the microscope can be adjusted to image all areas.

The conclusions drawn in this study were based on the assumption that the phantom used was homogeneous. This was supported by both the literature demonstrating IL to have the appropriate characteristics and the high intraclass correlation between phantom 1 and 2 [28,29]. However, one limitation of this study was the time difference between measurements. As ICG is susceptible to photobleaching, the ICG molecules could have lost fluorescence intensity over time. Since the center of the phantom was exposed to more light, the ICG molecules in the center may have lost their fluorescence abilities more than those in the periphery. This could potentially lead to an underestimation of the center–periphery effect, especially for the Tivato. However, we believe this effect was minimized by limiting illumination to just 10 s per measurement and storing the phantoms in darkness between each measurement. Furthermore, our photobleaching test showed only a 3% decrease in signal intensity, indicating a negligible quenching effect. Therefore, we consider it unlikely that photobleaching significantly influenced the study results. Additionally, the static recording of a phantom does not capture the dynamic properties of tissue flaps in vivo. Variables such as blood circulation, respiratory movements of the patient, and variations in perfusion across diverse tissue layers (such as skin and muscle) should also be acknowledged when evaluating NIRF images.

In conclusion, when using surgical microscope-integrated ICG-FA, the center–periphery effect must be taken into consideration, both in clinical practice and with the aim of quantifying tissue perfusion. Future studies may focus on the presence of this effect and how it could be corrected for when using objective measurements of fluorescence imaging. When the Zeiss microscope is used for fluorescence imaging, a setting of 30 cm in working distance and 3.5× magnification produces the most homogeneous videos. To further mitigate the center–periphery effect, the diameter and intensity of the light beam can be increased. Lastly, for image assessments and quantitative analyses, the central region of the FoV remains the most reliable and informative area. The center–periphery effect is a potential pitfall that a microsurgeon should be aware of, especially when a large field of view is needed for the assessment of the viability of a flap.

## Figures and Tables

**Table 1:** Absolute maximum fluorescence intensity (arbitrary unit 0–255) per setting in the Pentero 900, corrected with the dark measurement.

Pentero 900						
Working Distance	Magnification Factor					
	Min	3.5	5.5	7.5	10	Max
20 cm		144	144	135	123	61
30 cm	130	130	132	117	65	32
40 cm	71	151	153	100	58	40
50 cm	53	101	78	42		

**Table 2:** Absolute maximum fluorescence intensity (arbitrary unit 0–255) per setting in the Tivato 700, corrected with the dark measurement.

Tivato 700						
Working Distance	Magnification Factor					
	Min	3.5	5.5	7.5	10	Max
20 cm	215	214	214	214	200	79
30 cm	135	135	134	110	61	40
40 cm	84	84	81	44	25	
50 cm	56	56	39	81		
60 cm	34	33	14	6		

**Table 3:** Absolute maximum light intensity (AMI) (arbitrary unit 0–255) and coefficient of variation (CV) in different light intensity settings in several underexposed images using the Tivato 700. Working distance (WD), magnification factor (MF), and the phantom used are described per recording.

WD; MF		50% Light Intensity	100% Light Intensity
30 cm; 12.7× (phantom 2)	CV	0.181	0.177
	AMI	39.7	113.7
60 cm; 2.8× (phantom 2)	CV	0.64	0.64
	AMI	25.3	66.9
60 cm; 3.5× (phantom 2)	CV	0.398	0.367
	AMI	24.8	67.5
60 cm; 5.5× (phantom 1)	CV	0.284	0.254
	AMI	14.5	42
60 cm; 7.5× (phantom 1)	CV	0.244	0.177
	AMI	6.4	19.1
60 cm; 5.5× (phantom 2)	CV	0.303	0.263
	AMI	10.2	30.9
60 cm; 7.5× (phantom 2)	CV	0.2	0.172
	AMI	8.68	27.15

## Data Availability

The original data presented in the study are openly available in Figshare: https://doi.org/10.6084/m9.figshare.28329281.

## Abbreviations

The following abbreviations are used in this manuscript:

NIRF	Near-infrared fluorescence
ICG	Indocyanine green
ICG-FA	Indocyanine green fluorescence angiography
WD	Working distance
FoV	Field of view
MF	Magnification factor
PBS	Phosphate-buffered saline
FSL	Fluorescence signal loss
F_low_	Lowest fluorescent intensity
F_high_	Highest fluorescent intensity
AMI	Maximum light intensity
CV	Coefficient of variation

## Appendix A

**Table A1:** Field of view (FoV) per magnification.

Pentero		Tivato	
Magnification	FoV (mm) Horizontal × Vertical	Magnification	FoV (mm) Horizontal × Vertical
20	13 × 10	17.4	19 × 10
15.1	17 × 14	12.7	14 × 8
12.1	22 × 18	10	23 × 13
10	26 × 21	7.5	31 × 18
7.5	35 × 28	5.5	43 × 24
5.5	47 × 39	3.5	67 × 37
3.5	75 × 61	2.8	206 × 116
2.6	98 × 80	2.1	167 × 94
2.1	131 × 107	1.7	139 × 78
1.7	148 × 121	1.4	112 × 63
		1.1	82 × 46
